# The multi-protein family of sulfotransferases in plants: composition, occurrence, substrate specificity, and functions

**DOI:** 10.3389/fpls.2014.00556

**Published:** 2014-10-16

**Authors:** Felix Hirschmann, Florian Krause, Jutta Papenbrock

**Affiliations:** Institute of Botany, Leibniz University HannoverHannover, Germany

**Keywords:** *Arabidopsis thaliana*, glucosinolate, histidine residue, phosphoadenosine 5′-phosphosulfate, sulfotransferase

## Abstract

All members of the sulfotransferase (SOT, EC 2.8.2.-) protein family transfer a sulfuryl group from the donor 3′-phosphoadenosine 5′-phosphosulfate (PAPS) to an appropriate hydroxyl group of several classes of substrates. The primary structure of these enzymes is characterized by a histidine residue in the active site, defined PAPS binding sites and a longer SOT domain. Proteins with this SOT domain occur in all organisms from all three domains, usually as a multi-protein family. *Arabidopsis thaliana* SOTs, the best characterized SOT multi-protein family, contains 21 members. The substrates for several plant enzymes have already been identified, such as glucosinolates, brassinosteroids, jasmonates, flavonoids, and salicylic acid. Much information has been gathered on desulfo-glucosinolate (dsGl) SOTs in *A. thaliana*. The three cytosolic dsGl SOTs show slightly different expression patterns. The recombinant proteins reveal differences in their affinity to indolic and aliphatic dsGls. Also the respective recombinant dsGl SOTs from different *A. thaliana* ecotypes differ in their kinetic properties. However, determinants of substrate specificity and the exact reaction mechanism still need to be clarified. Probably, the three-dimensional structures of more plant proteins need to be solved to analyze the mode of action and the responsible amino acids for substrate binding. In addition to *A. thaliana,* more plant species from several families need to be investigated to fully elucidate the diversity of sulfated molecules and the way of biosynthesis catalyzed by SOT enzymes.

## INTRODUCTION

**FIGURE 1:**
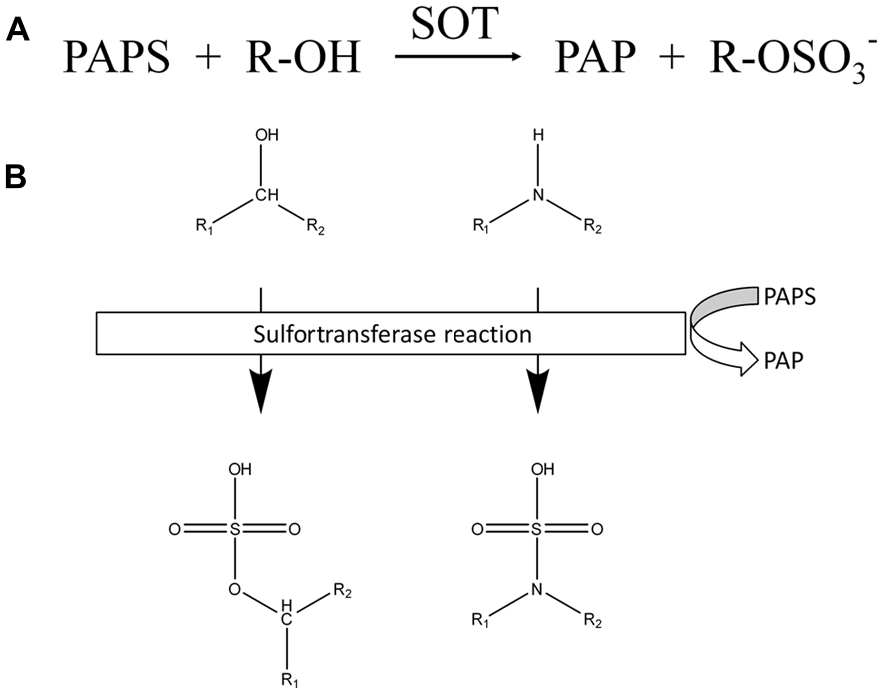
**Reactions catalyzed by SOTs. (A)** Chemical equation of reactions catalyzed by SOTs. **(B)** Schematic overview of SOT targets. Chemical structure of targeted hydroxyl and amide groups and their sulfated products, sulfate ester and the sulfamate group.

Members of the sulfotransferase (SOT) family have been found in all organisms investigated to date. All of these enzymes catalyze the transfer of a sulfuryl group from 3′-phosphoadenosine 5′-phosphosulfate (PAPS) to an appropriate hydroxyl group (**Figure 1**), hydroxyl amine or unprotonated amine of various substrates with the parallel formation of PAP.

The SOTs catalyze the sulfation of a wide range of compounds and produce sulfate esters, sulfamates, and sulfate conjugates (Klaassen and Boles, 1997). A sulfate conjugate is more water soluble than a non-sulfated molecule (Weinshilboum and Otterness, 1994), thus facilitating excretion and bioactivation.

Due to the unifying use of the co-substrate PAPS, all SOT proteins are characterized by a histidine residue in the active site, defined PAPS binding sites and a defined SOT domain (Pfam: PF00685; Finn et al., 2014). Proteins with this SOT domain occur in all organisms from all three domains investigated so far, usually as a multi-protein family. Originally, the SOT proteins in mammals were classified on the basis of their affinity for different classes of substrates. One group of SOT proteins, mainly membrane-associated, accepts as substrates macromolecules, such as proteins and peptides, and glycosaminoglycans (Niehrs et al., 1994). The second group, usually soluble proteins, accepts as substrates small organic molecules, such as flavonoids, steroids, and xenobiotics, with diverse chemical structures. In plants, the best criteria for forming subgroups within the multi-protein family is still a matter of debate, because either sequence identity/similarity or their substrate specificity could be chosen. Several compounds have been found in different plant species, such as: brassinosteroids, coumarins, flavonoids, gibberellic acids, glucosinolates (Gls), phenolic acids, sulfate esters such as choline-*O*-sulfate, and terpenoids that might be sulfated by SOT proteins. However, only for some of these substrates has the catalyzing SOT protein been identified. Not all sulfated compounds are necessarily sulfated by SOTs. Sulfolipids contain a 6-deoxy-6-sulfoglucose sugar head group, referred to as sulfoquinovose. The sulfoquinovose precursor UDP-sulfoquinovose is biosynthesized from UDP-glucose by a UDP-sulfoquinovose synthase associated with a ferredoxin-dependent glutamate synthase using sulfite as cosubstrate (Shimojima et al., 2005). Much information has been gathered on desulfo-glucosinolate (dsGl) SOTs in *Arabidopsis,* differing in their affinity to indolic and aliphatic dsGls. However, determinants of substrate specificity and the exact reaction mechanism still need to be clarified. Probably, the three-dimensional structures of more plant proteins have to be solved to analyze the mode of action and the responsible amino acids for substrate binding.

## PRIMARY STRUCTURE OF SOTs, PAPS BINDING REGIONS, AND ALIGNMENT OF THE HIGHLY CONSERVED REGIONS

Generally, SOTs can be divided into membrane-bound proteins and soluble cytosolic proteins. So far, only a few membrane-bound SOTs have been characterized in plants. They are either bound to the plasma membrane, as shown for the gallic acid glucoside SOT from *Mimosa pudica* L. (Varin et al., 1997a), or localized in the Golgi apparatus, as shown for the tyrosylprotein SOTs (TPSTs) from *Asparagus officinalis* L. (Hanai et al., 2000) and *Arabidopsis thaliana* (L.) Heynh. (Komori et al., 2009). The term cytosolic SOT might indicate a localization in the cytoplasm, yet the name implies that the proteins can be purified from plant cells and kept in solution (Hernàndez-Sebastiá et al., 2008). The exact localization of most cytosolic plant SOTs still remain unknown.

**FIGURE 2:**
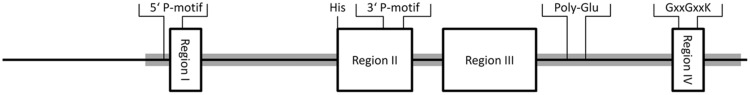
**Conserved regions I to IV of plant SOTs.** Regions are shown as boxes, with size and position in the protein relative to the average size of SOTs from *Arabidopsis thaliana*. Functional amino acids were obtained from structural analyses of mouse SOTs as described above. The PAPS binding regions (5′P-motif, 3′P-motif, GxxGxxK), the proton acceptor histidine (His), and a poly-glutamic acid (Poly-Glu), that can be found in many plant SOTs, are labeled. The position of the Pfam domain PF00685 is shaded in gray.

Sequence alignments of eleven cytosolic SOTs from plants, animals, and bacteria resulted in the identification of four highly conserved regions I to IV (Marsolais and Varin, 1995; **Figure 2**). Further analyses showed that especially the regions I and IV are highly conserved, for example throughout the SOT family of *A. thaliana* (Klein and Papenbrock, 2004). The regions I, II, and IV are responsible for the binding of the co-substrate PAPS (Varin et al., 1997b). The first structural approach to clarify the relevance of the regions for PAPS binding was determined by X-ray crystallography analyses of a mouse estrogen SOT (Kakuta et al., 1997). Region I is localized close to the N-terminus and includes the PAPS binding domain (PSB domain) that interacts with the 5′-phosphate of PAPS. Region II starts with a characteristic highly conserved histidine, responsible for proton acceptance during the sulfuryl transfer (Kakuta et al., 1998). In the C-terminal part of region II the two amino acids Arg130 and Ser138 are responsible for the binding of the 3′-phosphate of PAP and form a 3′ P-motif (Kakuta et al., 1997). This motif can be found in 18 SOTs out of 22 from *A. thaliana* and from almost all other plant SOTs. In many plant SOTs, a conserved hydrophilic site containing poly-glutamic acid (Poly-Glu) of unknown function can be found between region III and IV. Similar motifs have been found in a human chondroitin 6-SOT, but at a different position (Fukuta et al., 1998). Region IV is localized at the C-terminus and contains a P-loop related GxxGxxK motif (**Figure 2**).

In *A. thaliana*, 18 protein sequences with high similarity to known SOTs have been originally identified by BLAST approaches (Klein and Papenbrock, 2004). Later, another three SOTs were added (Klein and Papenbrock, 2008). These were formerly annotated in NCBI as “nodulation-related protein” and are now annotated as “P-loop containing triphosphate hydrolase family protein.” In addition to these SOTs, a TPST has been identified (Komori et al., 2009). Furthermore, a not yet literarily mentioned protein, Q9SCR3, with a *Sulfotransfer_1* domain (PF00685), is available in the Pfam database [http://pfam.xfam.org/protein/Q9SCR3 (accessed 23.06.2014)]. About 75% of the amino acid sequence is identical to *A. thaliana* SOT (AtSOT19). Therefore, it might be a redundant entry or a product of a different splicing process. Interestingly, AtTPST is exceptional in its structure compared to the remaining AtSOTs. With 500 amino acids, it is not only bigger, but is also the solely identified transmembrane, *cis*-Golgi localized AtSOT. Furthermore, it shows no sequence similarity to human TPSTs; and no other typical features like the regions I to IV and the characteristic highly conserved histidine were identified (Komori et al., 2009). It is also the only *Arabidopsis* SOT that contains a *Sulfotransfer_2* domain (PF03567), instead of a *Sulfotranfer_1* domain (PF00685). Hence, it is only associated by function and not by sequence. Excluding the pseudogenic sequence *AtSOT2* and TPST, the SOT protein lengths range between 273 and 403 amino acids with an average length of 321 amino acids. Only seven out of 21 *AtSOTs* contain introns.

There are several nomenclatures for *A. thaliana* SOTs used in the literature. The most common ones are listed in **Table 1**, including information about the preferred SOT substrates. In this review, the nomenclature first introduced by Klein and Papenbrock (2004) is used.

**Table 1:** Summary of the members of the SOT family in *Arabidopsis* and their putative substrates.

		Nomenclature					
NCBI accession	*Arabidopsis* gene ID	I	II	III	Amino acids	Preferred substrate	Reference
NP_199182	AT5G43690	AtSOT1		AtSULT202B4	331		
NP_190689	AT3G51210	AtSOT2		Pseudogene	67		
NP_194358	AT4G26280	AtSOT3		AtSULT202C1	314		
NP_180325	AT2G27570	AtSOT4		AtSULT202B3	273		
NP_190093	AT3G45070	AtSOT5	AtST3a	AtSULT202B1	323	Flavonol	Gidda and Varin (2006), Hashiguchi et al. (2013)
NP_190094	AT3G45080	AtSOT6	AtST3b	AtSULT202B2	329		
NP_174139	AT1G28170	AtSOT7		AtSULT202B8	326		
NP_172799	AT1G13420	AtSOT8	AtST4b	AtSULT202B7	331	Flavonol glycosides	Hashiguchi et al. (2014)
NP_172800	AT1G13430	AtSOT9	AtST4c	AtSULT202B5	351		
NP_179098	AT2G14920	AtSOT10	AtST4a	AtSULT202B6	333	Brassinosteroids	Marsolais et al. (2007)
NP_565305	AT2G03750	AtSOT11		AtSULT202D1	351		
NP_178471	AT2G03760	AtSOT12	AtST1	AtSULT202A1	326	Flavonone, brassinosteroids, salicylic acid	Lacomme and Roby (1996), Marsolais et al. (2007), Baek et al. (2010), Hashiguchi et al. (2013)
NP_178472	AT2G03770	AtSOT13		AtSULT202E1	324	Flavonol	Hashiguchi et al. (2013)
NP_196317	AT5G07000	AtSOT14	AtST2b	AtSULT203A2	347		
NP_568177	AT5G07010	AtSOT15	AtST2a	AtSULT203A1	359	Hydroxyjasmonate	Gidda et al. (2003)
NP_177550	AT1G74100	AtSOT16	AtST5a	AtSULT201B3	338	Phenylalanine and tryptophan derived dsGls	Piotrowski et al. (2004), Klein et al. (2006)
NP_173294	AT1G18590	AtSOT17	AtST5c	AtSULT201B2	346	Benzyl and methionine derived dsGls	Piotrowski et al. (2004) Klein et al. (2006)
NP_177549	AT1G74090	AtSOT18	AtSTb	AtSULT201B1	350	Phenylalanine and methionine derived dsGls	Piotrowski et al. (2004), Klein et al. (2006), Luczak et al. (2013)
NP_190631	AT3G50620	AtSOT19			340		
NP_179175	AT2G15730	AtSOT20			344		
NP_195168	AT4G34420	AtSOT21			403		
NP_563804	AT1G08030	TPST			500	Tyrosylprotein	Komori et al. (2009)

*I: Nomenclature used in this review introduced by Klein and Papenbrock (2004). II: Nomenclature introduced by Piotrowski et al. (2004). III: Nomenclature introduced by Hashiguchi et al. (2013). The term TPST was introduced by Komori et al. (2009).*

So far, only one SOT from plants (AtSOT12 from *A. thaliana*) was structurally solved (Smith et al., 2004). Therefore, most SOT proteins lack structural analyses and detailed enzymological characterizations. The identified motifs only give a hint on the proteins’ general function as a SOT, but no information about their specificity and affinity toward certain substrates.

Most proteins identified as putative SOTs contain at least one out of seven related Pfam motifs that are based on Hidden Markov Models (HMM). The most important HMMs referring to SOTs are the SOT domains *Sulfotransfer_1* (PF00685), *Sulfotransfer_2* (PF03567), and *Sulfotransfer_3* (PF13469), which have an average length of 230.1, 218.3, and 224 amino acids, respectively. According to the model information in the Pfam database, the SOT domain 1 shows an average coverage of its contributing protein sequences of 64%. An average of 16% of all amino acid residues that are covered by the HMM are identical to it. The average coverage of the SOT domains 2 and 3 in their respective sequences are 67 and 47%, with average sequence identities of 15 and 14%. In addition, HMMs for more specific SOT subfamilies have been deposited in Pfam. Generally, they show a lower number of hits in the database, with mostly increased sequence coverage and a higher average sequence identity as compared to the more general SOT domains 1–3. The aryl SOT domains *Arylsulfotrans_1* and *Arylsulfotrans_2* (PF05935 and PF14269) and the Stf0 SOT domain *Sulphotransf* (PF09037) represent groups of sequences with more specific occurrence, especially in prokaryota. The two aryl SOT domains show an average coverage of 83 and 57%, with an identity of 30 and 28%, respectively. For the Stf0 SOT domain the average sequence coverage is 81%, with an average identity of 33%. The galactose-3-*O*-SOT domain (PF06990) shows coverage of 78% with 24% identity.

## SULFOTRANSFERASE FAMILIES IN DIFFERENT PLANT GENOMES

Sulfotransferases have a broad range of substrates and therefore many functions. In previous literature it has been stated that SOTs are present in all kingdoms except in Archaea (Klein and Papenbrock, 2008; Chen et al., 2012). Nevertheless, according to the protein family database Pfam [http://pfam.xfam.org/ (accessed 23.06.2014)] there are Archaea sequences with a characteristic conserved SOT domain.

There are only few studies that aim to identify all SOTs of a plant species. A requirement to do this is a fully sequenced genome, but due to their eclectic functions, we assume that SOTs are present in almost every plant species. The Pfam database already stores 538 putative plant SOT sequences, 459 of which have a *Sulfotransfer_1* domain (PF00685), 49 a *Sulfotransfer_2* domain (PF03567), 16 a *Sulfotransfer_3* domain (PF13469), and 10 a *Sulphotransf* domain (PF09037). The actual number might be less, because of redundant entries. The *Sulfotransfer_3* (PF13469) and the *Gal-3-O_sulfotr* domain (PF06990) are only present in algae. The *Arylsulfotrans* domain (PF05935) is not present in plants, while *Arylsulfotran_2* (PF14269) is only found in a single *Ricinus communis* L. sequence.

While so far 22 putative *A. thaliana* SOTs were identified, 35 genes coding proteins with a SOT domain were reported in *Oryza sativa* L., including six genes likely to be pseudogenes (Chen et al., 2012). In phylogenetic analyses, they are clustered into seven subfamilies. However, microarray data revealed that the genes within subfamilies are expressed in a different manner, indicating individual functions. When 17 AtSOTs were added to the distance trees, they did not group together with any of the *O. sativa* genes. This was taken as a hint for independent evolution of *O. sativa* and *A. thaliana* SOTs by gene duplication or loss. This was supported by the finding that half of the *O. sativa SOTs* contain introns, which is hardly the case for *AtSOTs* (Klein and Papenbrock, 2004).

Comparative genomics studies were conducted in *Brassica rapa* L. with *A. thaliana*, in order to identify all Gl biosynthesis genes (Zang et al., 2009; Wang et al., 2011). Thirteen putative *desulfo-glucosinolate SOT* (*dsGl SOT*) genes were identified. Two genes are paralogs of *AtSOT16*, 1 of *AtSOT17*, and 10 of *AtSOT18*. One *AtSOT18* paralog appears to be nonfunctional, because of transposon insertion, and one carries a frame shift. None of the genes contains introns, as it is the case for *AtSOT* genes. All paralogs share at least 70% sequence identity with their *AtSOT* counterparts, with the exception of one SOT from *B. rapa* (*BrSOT18,* 68%; Wang et al., 2011). The higher number of *BrSOTs* is explained by the triplication of the *B. rapa* genome and later duplication, transposition, or tandem duplication of the genes.

In *Brassica napus* L., so far only twelve putative genes encoding SOTs were identified (Rouleau et al., 1999; Marsolais et al., 2000). Additionally, there are at least five isoforms of dsGl SOTs in *B. napus*, which have similar substrate affinities as their *A. thaliana* homologs (own unpublished results). Regarding that *B. napus* is an allotetraploid species formed by the hybridization of *B. rapa* and *B. oleracea*, a much higher number of *SOT* genes can be expected.

To group these diverse enzymes into families and subfamilies remains a difficult task. Klein and Papenbrock (2004, 2008) ordered 21 *A. thaliana* SOTs in eight groups, according to their amino acid sequence identity. However, already characterized SOTs with the same substrate specificity did not group together, and even high sequence identity of more than 85% among two SOTs did not reveal equal enzymological characteristics. Neither sequence identity, nor generated trees ordered already characterized SOTs in groups according to their substrate specificities. Three dsGl SOTs were on one separate branch, but flavonoid and brassinosteroid SOTs could not be distinguished.

Hernàndez-Sebastiá et al. (2008) generated a phylogenetic tree including 78 SOTs from 13 different plant species. This approach faced the same problems as the one by Klein and Papenbrock (2004, 2008) and it was again concluded that the prediction of SOT substrates by high primary sequence identities is limited. For example, it was speculated that AtSOT13 was a brassinosteroid SOT, because of its close distance to AtSOT12, but Hashiguchi et al. (2013) showed that AtSOT13 uses flavonoids as preferred substrates.

Another attempt included, besides 17 *A. thaliana,* also *B. napus* and *Flaveria* spp. sequences (Hashiguchi et al., 2013). *AtSOT2* was excluded, because it is most likely a pseudogene. According to a dendrogram, three families were defined with two, three and five subfamilies, respectively. The families had an amino acid sequence identity of at least 45% and the subfamilies of at least 60%. But again, except for the dsGl SOTs, the SOTs did not group together according to their substrate specificities.

Labonne et al. (2009) tried to identify a putative *SOT* of *Turnera krapovickasii* Arbo (Passifloraceae) by phylogenetic analysis. The sequence was aligned with 28 SOTs from *A. thaliana*, *B. napus*, *Vitis vinifera* L., *O. sativa*, *Hordeum vulgaris* L., *Populus trichocarpa* Hook. The SOT from *T. krapovickasii* was on a branch by itself and alignments with characterized SOTs revealed low sequence identity. Therefore, it was not possible to identify the function of the respective SOT.

Overall, past attempts indicate that it is difficult to order plant SOTs according to their amino acid sequence. Only for dsGl SOTs does it seem to be possible, because they are clustered together on a separate branch in all approaches. Therefore, only enzymatic assays with additional mutational studies can give reliable information about substrate specificity and function.

## SUBSTRATES FOR SULFOTRANSFERASES

### BIOSYNTHESIS OF THE CO-SUBSTRATE PAPS

**FIGURE 3:**
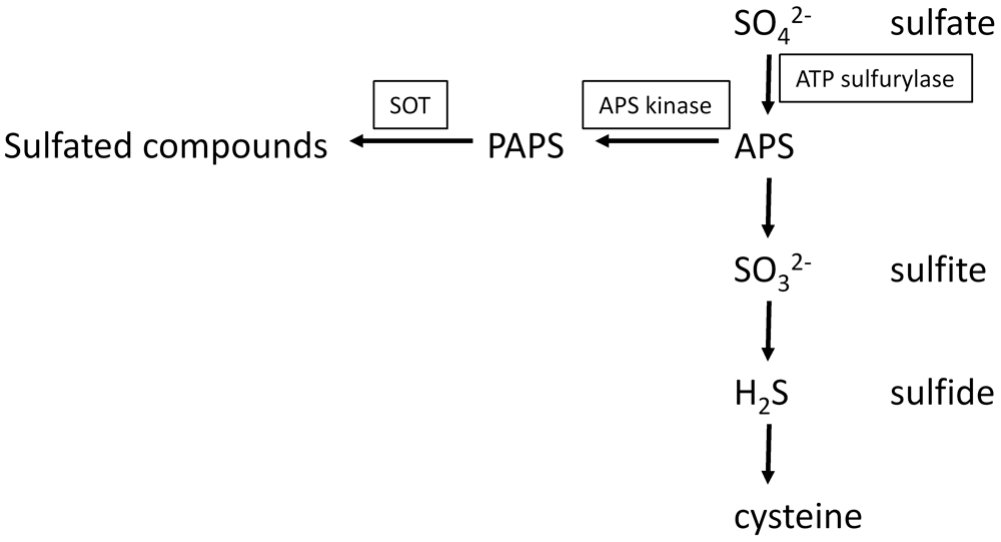
**Biosynthesis of APS and PAPS and the connection of primary and secondary sulfur metabolism**.

3′-phosphoadenosine 5′-phosphosulfate is an obligate co-substrate for sulfation reactions catalyzed by SOTs. In plants, PAPS does not represent an intermediate of reductive sulfate assimilation as in fungi and some bacteria, but it seems to play an exclusive role as a sulfuryl donor for sulfation reactions. PAPS is synthesized from ATP and sulfate in a two-step reaction (**Figure 3**). In the first step, ATP sulfurylase (EC 2.7.7.4) catalyzes sulfate activation. The enzyme hydrolyses the bond between the β- and the γ-phosphates of ATP and then adds sulfate to the γ-phosphate. The activation step is necessary, because sulfate is metabolically inert. The energy is stored in the phosphoric acid-sulfuric anhydride bond of the reaction product, adenosine 5′-phosphosulfate (APS), allowing sulfate to undergo further reactions. The energetic balance of the sulfate adenylylation reaction favors ATP formation. Therefore, the reaction products, APS, and pyrophosphate (PP_i_), need to be maintained at a low concentration by the enzymes inorganic pyrophosphatase that hydrolyses PP_i_, APS reductase (EC 1.8.4.9) and APS kinase (EC 2.7.1.25; AKN) that metabolize APS. APS reductase catalyzes the first step of sulfate reduction. APS kinase catalyzes the ATP-dependent phosphorylation on the 3′-position of APS. *In vitro* tests have shown that excess APS inhibits APS kinase. The product PAPS is the substrate for the SOT proteins.

In general, the availability of PAPS for sulfation *in vivo* depends on its synthesis, transport, degradation, and utilization as investigated in mammals (Klaassen and Boles, 1997). Recently, it was shown that the transporter PAPST1 in the chloroplast envelope membrane is not only involved in the provision of PAPS for the extraplastidic sulfation reactions, but is also capable to transport PAP in an antiport manner. The loss of *PAPST1* leads to a decreased production of sulfated compounds like Gl, increased production of dsGl, and the modulation of primary sulfate assimilation, another indication for the strong interconnectedness of primary and secondary sulfur metabolism (Gigolashvili et al., 2012). The by-product of the sulfation reaction, PAP, has gene regulatory attributes. In turn, PAP is regulated by the adenosine bisphosphate phosphatase SAL1 that dephosphorylates PAP to adenosine monophosphate. 3′-Phosphoadenosine 5′-phosphate accumulates at drought stress and high light conditions. Mutational studies indicated that PAP inhibits 5′–3′ exoribonucleases in the cytosol and nucleus, which causes changes in expression of stress-responsive genes. It was suggested that a PAP-SAL1 retrograde pathway alters gene expression as part of the stress response (Estavillo et al., 2011). Additionally, a correlation between the increases of PAP with changes in the sulfur metabolism was reported. Further analysis of *sal1* knock out mutants led to the conclusion that changes of gene expression due to sulfur limitation is triggered by internal sulfur deficiency and not by low external sulfur levels. PAP accumulation also resulted in an increase of enzymatic oxygenation of fatty acids, an increase of jasmonic acid synthesis and a decrease of Gls. Possible explanations for the Gl decrease could be inhibition of dsGl SOTs or the disruption of PAPS transport from plastids to the cytosol (Gigolashvili et al., 2012; Lee et al., 2012).

### SUBSTRATES FOR PLANT SOTs

The first isolated and characterized plant SOTs were flavonol 3′- and flavonol 4′-SOT of *Flaveria chloraefolia* (Varin et al., 1992) and later of *F. bidentis* (L.) Kuntze (Varin et al., 1997b). These SOTs sequentially sulfate specific hydroxyl groups of the flavonol quercetin to quercetin tetrasulfate. Flavonol biosynthesis was demonstrated to be regulated by auxin and ethylene. In turn, the flavonol quercetin and quercetin sulfates affect root development processes, such as the basipetal root auxin transport, elongation growth, and gravitropism (Faulkner and Rubery, 1992; Lewis et al., 2011).

So far, four flavonoid SOTs have been characterized in *A. thaliana*: AtSOT5, AtSOT8, AtSOT12, and AtSOT13 (see **Table 1** for details). Hashiguchi et al. (2013) compared characteristics and substrate specificities of AtSOT5, AtSOT12, and AtSOT13.

AtSOT13 and AtSOT5 showed the highest activity with the flavonol galangin (3,5,7-trihydroxy-2-phenyl-4H-chromen-4-one), while AtSOT12 showed the highest activity for the flavanone naringenin [(2S)-5,7-dihydroxy-2-(4-hydroxyphenyl)-2,3-dihydro-4H-chromen-4-one] and was the only SOT that sulfates anthocyanidin. Interestingly, the AtSOTs showed no or comparably low activity with quercetin. It was speculated that the position-3 hydroxyl group of quercetin inhibits the catalytic activity. It was also shown that only AtSOT12 is able to use 3-hydroxyflavone as substrate, while 7-hydroxyflavone is used by all three AtSOTs. After comparisons of *K*_m_-values using kaempferol as substrate, it was concluded that particular hydroxyl groups of kaempferol are specifically sulfated by the AtSOTs.

AtSOT8 was also characterized by Hashiguchi et al. (2014). The pH optimum at 5.5 was lower than for previously characterized flavonoid SOTs. Thus it was speculated that AtSOT8 might be located in the vacuole. Comparison of *V*_max_/*K*_m_-values showed that AtSOT8 prefers flavonol glycosides instead of their aglycone counterparts as substrates. Also, there was only activity to flavonoids with a hydroxyl group at position 7. Hence, it was suggested that AtSOT8 might be a flavonol glucoside-7 SOT. Surprisingly, neither sulfated glucoside flavonoids could be detected *in vivo* in *A. thaliana* by LC/MS, nor were there any accordant database entries. Possible explanations for the non-detected sulfated glucoside flavonoids could be low or condition-dependent occurrence.

AtSOT10 showed activity with brassinosteroids (Marsolais et al., 2007), specifically brassinosteroid biosynthetic end products. In summary, it was speculated that it inactivates brassinosteroids and therefore is involved in plant development processes. In numerous studies, overexpression of brassinosteroid catabolic genes led to brassinosteroid-deficient phenotypes. However, overexpression and T-DNA insertion in null mutants of AtSOT10 did not show brassinosteroid-deficient phenotypes, emphasizing difficulties of transferring *in vitro* results to *in vivo* insights (Sandhu and Neff, 2013).

Of all investigated *A. thaliana* SOTs so far, AtSOT12 has the broadest substrate specificity. Besides using flavonoids as substrates, it was also shown to be active with brassinosteroids and salicylic acid. Within the brassinosteroids, it showed preference for 24-epibrassinosteroids (Marsolais et al., 2007). It was stereospecific for 24-epibrassinosteroids and accepted mammalian hydroxysteroids and estrogens, too. The most preferred substrate was the metabolic precursor 24-epicathasterone (*K*_m_ = 6.9 μM), which showed inhibitory effects above 5 μM. The *K*_m_-value for salicylic acid is comparably high (440 μM; Baek et al., 2010). Salicylic acid is a signal molecule in plant defense, and cellular concentrations increased up to 40 μM after pathogen infection indicating that sulfation of salicylic acid is a response to pathogen attack. This theory was supported by the fact that *atsot12* knock out mutants were less resistant to the pathogen *Pseudomonas syringae*, while AtSOT12 overexpressing lines showed a higher resistance.

Two *B. napus* brassinosteroid isoforms, BNST3 and BNST4, were enzymatically characterized. Recombinant BNST3 stereospecifically sulfated 24-epibrassinosteroids and preferred 24-epicathasterone (K_m_ = 1.4 μM), which is a biosynthetic intermediate of 24-epibrassinolide. Because of the biological inactivity of 24-epibrassinolide sulfate, it was hypothesized that BNST3 is involved in brassinosteroid inactivation (Rouleau et al., 1999). BNST4 also preferred 24-epibrassinosteroids (K_m_ = 4.9 μM), but also showed a broad substrate specificity with other steroids, also indicating a role in detoxification (Marsolais et al., 2004). Overall, they showed similar substrate specificities toward brassinosteroids as AtSOT12 (Marsolais et al., 2007).

AtSOT15 specifically sulfates 11- and 12-hydroxyjasmonate, which is a signaling molecule in plant defense and development. *K*_m_-values indicate a higher affinity to 12- than to 11-hydroxyjasmonate (10 μM and 50 μM, respectively). 12-hydroxyjasmonate naturally occurs in *A. thaliana* and it was suggested that sulfation might function in inactivation of 12-hydroxyjasmonic acid (Gidda et al., 2003).

Komori et al. (2009) identified a 62 kDa, Golgi-localized, transmembrane protein, that sulfates tyrosylproteins in *A. thaliana*. The recombinantly expressed TPST sulfated tyrosine residues of precursor polypeptides of the “plant peptide containing sulfated tyrosine 1” (PSY1) and phytosulfokine (PSK). PSY1 and PSK are peptide hormones, which promote growth and cell proliferation (Matsubayashi and Sakagami, 1996; Amano et al., 2007). The activity with both substrates indicates broad substrate specificity. TPST showed a higher activity with PSY1, which was explained by a closer proximity of an acidic region to the sulfated tyrosine residue. *TPST* loss-of-function mutants showed numerous abnormal attributes, which led to the conclusion that sulfated peptides or proteins are involved in plant growth and development. Previously, in microsomal membrane preparations from carrot cells, rice, and asparagus TPST activity was shown (Hanai et al., 2000). In rice, the *K*_m_-value was 71 μM at a pH of 7.0–8.5 in the presence of manganese ions. The enzyme kinetic values such as *K*_m_ and *V*_max_ *A. thaliana* TPST remain to be determined. TPST also sulfates peptide root meristem growth factors (RGFs), which are involved in postembryonic root development. Loss-of-function *tpst-1* mutants showed reduction in root meristem size and loss of coordination between cell elongation and expansion in the elongation–differentiation zone. Addition of RGF restored the meristem activity to ∼70% and addition of RGF, PSK, and PSY1 restored the activity comparable to the wild-type. Sulfation of RGFs was found to be critical for its function. Further experiments showed that RGFs positively regulate the expression of PLETHORA transcription factors that mediate the pattern of the root stem niche (Matsuzaki et al., 2010).

Other examples of already characterized SOTs like a choline-*O*-sulfate SOT of the halophytic *Limonium* species (Rivoal and Hanson, 1994) and a plasma membrane-associated gallic acid SOT of *M. pudica* L. (Varin et al., 1997a) undercut the diversity of substrates and functions of these enzymes. Choline sulfate is an osmolyte that accumulates under saline conditions. The respective choline-*O*-SOT showed a fourfold higher activity under high salinity. The choline-*O*-SOT had its pH optimum at 9.0 and the *K*_m_-value for choline was 25 μM. The 42 kDa membrane bound gallic acid SOT might be involved in the regulation of the seismonastic response. It showed strict substrate specificity and a K_m_-value of only 3.0 μM.

Further studies indicate the existence of more SOTs, even though they were not especially isolated or characterized. It was shown *in vivo* that poplar trees convert hydroxylated metabolites of polychlorinated biphenyls (PCBs) into sulfated PCB. It was suggested that SOTs catalyze this reaction (Zhai et al., 2013). Sulfated polysaccharides occur in marine angiosperms, mangroves (Aquino et al., 2005, 2011), freshwater plants (Dantas-Santos et al., 2012), and algae (Ngo and Kim, 2013), which are likely to be sulfated by not yet identified SOTs.

### GLUCOSINOLATES: PRODUCTS OF THE SOT REACTION

Glucosinolates are a group of over 200 nitrogen- and sulfur-containing natural products found in vegetative and reproductive tissues of 16 plant families within the Capparales (Clarke, 2010). They are well-known as the major secondary metabolites in agriculturally important crop plants of the Brassicaceae family, such as oilseed rape (*B. napus*), fodder and vegetables (e.g,. broccoli and cabbage). The model plant *A. thaliana*. Gl share a core structure containing a β-D-glucopyranose residue linked via a sulfur atom to a (*Z*)-*N*-hydroximino sulfate ester. They are distinguished by a variable R group derived from one of several amino acids, mainly tryptophan, phenylalanine and methionine (Mithen, 2001). The Gl pattern varies among the plant species and among *A. thaliana* ecotypes. In 39 *A. thaliana* ecotypes, 34 different Gls have been identified. Quantity and composition of Gls depend on the developmental stage of the plants and on the plant organ (Kliebenstein et al., 2001a).

Intact Gls are not toxic to cells. However, after cell damage Gls are hydrolyzed, catalyzed by thioglucosidase enzymes (“myrosinase”), to produce a variety of volatile hydrolysis products, such as thiocyanates, isothiocyanates, and nitriles. Only these breakdown products have a wide range of biological activities including both negative and positive effects (Fenwick and Heaney, 1983). In several studies these breakdown products were shown to be involved in plant defense against pathogens and herbivores. Thus, Gls are the best-characterized preformed defense compounds in the Brassicaceae and contribute to the protection against pathogens of the generalist type (Rausch and Wachter, 2005).

The last step in the Gl core structure biosynthesis of the different aliphatic, aromatic, and indole desulfo (ds) Gls is catalyzed by members of the SOT family. Glendening and Poulton (1990) partially purified a protein from *Lepidium sativum* L. that had PAPS-dependent dsGl SOT activity; however, at that time no molecular data was available. Later it was shown that three SOT proteins from *A. thaliana* are involved in Gl biosynthesis catalyzing the sulfation of dsGls to the intact Gls (Varin and Spertini, 2003; Piotrowski et al., 2004; Hirai et al., 2005).

#### Sulfotransferases involved in sulfation of desulfo-glucosinolates

The three dsGl AtSOT proteins (AtSOT16, AtSOT17, and AtSOT18) were predicted and then verified by different means (screening of many sulfated compounds, combining of knowledge, and integration of metabolomics and transcriptomics) for being responsible for the sulfation of dsGl (Varin and Spertini, 2003; Piotrowski et al., 2004; Hirai et al., 2005). Up till now, it was not unambiguously demonstrated why multiple *dsGl SOT* genes have been conserved during evolution in *A. thaliana* and in other Brassicaceae species.

The glucosylation and the sulfation reactions were assumed to be non-specific with respect to the side chain (Halkier, 1999). It is also hypothesized that first the side chains are elongated to synthesize so-called parent Gls, then the glycone moiety is developed and finally, several side chain modifications take place to produce the respective daughter Gl (Wittstock and Halkier, 2002). However, it is not clarified when the dsGls are sulfated by SOT proteins and whether there is a specificity for certain parent or daughter Gls. As the Gl pattern differs among *A. thaliana* ecotypes (Kliebenstein et al., 2001a), the investigation of the three dsGl SOTs from ecotype C24, which shows the broadest variety of Gls in comparison to other ecotypes, was most rational. In addition, one exemplary SOT from the fully sequenced ecotype Col-0 was investigated. To determine if and how these three dsGl SOT proteins might influence the Gl pattern, different *in vitro* enzyme assays were performed. Substrate specificity varies among the three proteins in the same ecotype (C24) and between ecotypes (C24 versus Col-0). AtSOT16 (C24) has the broadest substrate specificities. Tryptophan and phenylalanine-derived dsGl are the most preferred substrates, but it also accepts methionine-derived dsGl of chain length C3, C4, C5, C6, C7, and C8, although at much lower activities. AtSOT17 (C24) has narrow substrate specificities and does not act upon tryptophan-derived dsGl. Phenylalanine-derived benzyl dsGl is the most preferred substrate, but it also accepts methionine-derived dsGls, but has a strong preference for longer side chains, C6, C7, and C8. AtSOT18 (C24) also has narrow substrate specificities, does not act upon tryptophan-derived dsGl. It accepts phenylalanine-derived dsGl and methionine-derived dsGl, but has a strong preference for longer side chains, C6, C7, and C8. In summary, these three enzymes differ significantly in their affinity for the investigated substrates and the co-substrate PAPS (Klein et al., 2006; Klein and Papenbrock, 2009). It was speculated that the differences between *AtSOT16-18* could be an explanation for the different Gl patterns between organs, developmentally stages and growth environments reported by Brown et al. (2003). Anyhow, Møldrup et al. (2011) transformed genes involved in Gl biosynthesis into tobacco, thus successfully enabling it to synthesize Gls. In this approach, they could show that SOTs are not the bottleneck of Gl synthesis, but the supply of the co-substrate PAPS. Therefore, regulation of AtSOT16–18 could be, only taken together with other Gl and PAPS biosynthesis genes, partly responsible for Gl variation.

Up to now, the knowledge on secondary modifications of parent Gls is limited (Graser et al., 2001). In future work it could be interesting to verify the general acceptance that parent dsGls are sulfated before secondary modifications of Gls take place (Kliebenstein et al., 2001b). However, assuming the general acceptance is right, no secondarily modified Gls would exist in a ds form to interact with the SOTs. Therefore, it is possible, that artificially de-sulfated Gls with secondary modifications are sulfated *in vitro*, but with no *in vivo* relevance.

#### In A. thaliana ecotypes SOT18 proteins differ in their sequence and substrate specificity

It was shown that AtSOT18 proteins from two different *A. thaliana* ecotypes differ in their kinetic parameters as well as their substrate specificities. The primary structure of AtSTO18 proteins from the ecotypes Col-0 and C24 differ in two amino acids (Klein et al., 2006; Klein and Papenbrock, 2009). One could assume that there could be a correlation of AtSOT18 enzyme activities and differences in Gl profiles between these ecotypes. Therefore, AtSOT18 sequences from eight *A. thaliana* ecotypes with highly diverse Gl patterns were investigated: The AtSOT18 sequence from Col-0 showed the highest similarity to the largest number of other sequences in the alignment. The AtSOT18 proteins showed sequence deviations of maximal two amino acids in comparison to the AtSOT18 sequence from Col-0. The positions of the amino acid replacements were different in each sequence. The small differences in the primary sequence lead to important structural changes in secondary and tertiary structure that might be the key for different kinetic activities toward a broad range of substrates (Luczak et al., 2013). All recombinant AtSOT18 proteins showed low substrate specificity with an indolic Gl, while the specificity for aliphatic substrates varied. There was no correlation in the kinetic behavior with the major dsGl contents or with the ratio of C_3_/C_4_ dsGl in the respective ecotype. Therefore, it is unlikely that dsGl AtSOT18 enzymes play a major role in shaping the Gl profile in *A. thaliana* (Luczak et al., 2013). Interestingly, in humans, inter-individual variation in sulfation capacity may be important in determining an individual’s response to xenobiotics, and recent studies have begun to suggest roles for SOT polymorphism in disease susceptibility (Gamage et al., 2006). Variations in concentration and composition of Gls in *A. thaliana* ecotypes and different environmental conditions can still neither be fully explained, nor predicted.

## EXPRESSION OF SULFOTRANSFERASES

Sulfated compounds are mainly linked to biotic and abiotic stress response. This is supported by several expression studies of *SOTs*. So far, the mRNA levels of most characterized *A. thaliana SOTs* were rather low under normal growth conditions (Lacomme and Roby, 1996; Gidda et al., 2003; Piotrowski et al., 2004). This is supported by the fact that there is a relatively low number of *SOTs* in EST databases with the exception of *AtSOT15* and *AtSOT16* (Klein and Papenbrock, 2008). However, the expression of *AtSOT12*, *AtSOT15*, *AtSOT16*, and *AtSOT17* was significantly increased by treatment with jasmonate (Lacomme and Roby, 1996; Gidda et al., 2003; Piotrowski et al., 2004).

Only 17 *AtSOTs* were found to be present on 24 k Affymetrix chips and for many of those the absolute signal was quite low (Klein and Papenbrock, 2008). Hashiguchi et al. (2013, 2014) reported that microarray database research suggested that *AtSOT8* is mainly expressed in roots, while At*SOT13* is expressed in the early stages of the embryonic development. Interestingly, Hashiguchi et al. (2013) cloned *AtSOT13* from 2-week old seedlings.

Transcripts of *AtSOT10* were mainly detected in roots. Transcript levels were repressed 4 h after *trans*-zeatin treatment. After 8 h no transcripts were detectable anymore by qRT-PCR (Marsolais et al., 2007).

Northern Blot analysis revealed that *AtSOT12*, the encoded protein uses brassinosteroids, flavonoids, and salicylic acid as substrates, is moderately expressed in roots and leaves, and highly in flowers, while no expression was detected in stems and siliques. Furthermore, it was strongly induced by salt and sorbitol and slightly by cold, ABA, auxins, cytokinins, methyl jasmonate, salicylic acid, and interactions with bacterial pathogens (Lacomme and Roby, 1996; Baek et al., 2010). These results strongly indicate a function of *AtSOT12* in stress and hormone response. Similar results were obtained for the respective homologous genes in *B. napus*. *BNST3* and *BNST4* mRNA levels were quite low, but increased after treatment with salicylic acid, ethanol, xenobiotics, low oxygen stres, and the herbicide safener naphtalic anhydride (Rouleau et al., 1999; Marsolais et al., 2004). *BNST3* and *BNST4* induction also indicates a function in stress response and detoxification.

The protein encoded by *AtSOT15* uses hydroxyl jasmonate as substrate. Expression was induced upon methyljasmonate and 12-hydroxyjasmonate treatment. Probably, it inactivates the function of jasmonic acids and therefore enhances the hypocotyl growth (Gidda et al., 2003). Yamashino et al. (2013) showed that *AtSOT15* transcription is also regulated by an external coincidence mechanism. Database research indicated that *AtSOT15* might be a target of the phytochrome interacting transcriptional factors PIF4 and PIF5. PIF4 and PIF5 are controlled by the circadian clock, but also independently influenced by light and temperature. Further qRT-PCR analysis showed that *AtSOT15* was diurnally regulated by PIF4 and PIF5 at the end of a short day dark phase and/or high temperatures. Accordingly, *AtSOT15* is induced under conditions when hypocotyl growth takes places.

At first, the dsGl SOTs *AtSOT16-18* were reported to be constitutively expressed in all leaves, flowers, and siliques (Varin and Spertini, 2003). Later Northern Blot analysis revealed that *AtSOT16* mRNA level increased after treatment with coronatine (an analog of octadecanoid signaling molecules), jasmonic acid precursor 12-oxophytodienonic, ethylene precursor ACC and after treatment with jasmonic acid. UV-C illumination and wounding also induced *AtSOT16* expression. *AtSOT17* mRNA increased 2.4 fold and 1.2 fold, respectively, while *AtSOT17* expression only slightly increased (1.3 fold) after coronatine treatment (Piotrowski et al., 2004). Regarding the developmental stages, *AtSOT16* and *AtSOT17* mRNA levels were highest in two week old seedlings and lowest in flowering plants. In contrary, *AtSOT18* levels were quite low in young plants and slightly increased after 5–6 weeks. Only *AtSOT17* expression was influenced by a 12 hour dark / 12 hour light cycle. It was the highest at the end of the light phase and the lowest at the end of the dark phase. No differences in any of the three mRNA levels were detected, when *A. thaliana* was grown in media with tenfold sulfate concentration (Klein et al., 2006).

Huseby et al. (2013) investigated how the Gl biosynthesis is controlled by light and the diurnal rhythm. By qRT-PCR analyses, it was shown that *AtSOT16*, *AtSOT17*, and *AtSOT18* are up regulated in light and down regulated in darkness. Further experiments indicated that the three *dsGl AtSOTs* are controlled by different transcriptional factors. In *A. thaliana* mutants, lacking the transcription regulator HY5, *AtSOT18* was less up-regulated than in the wild-type, indicating the HY5 is in control of *AtSOT18*. Interestingly, HY5 not only promotes numerous genes, but also seemed to repress MYBs. MYBs are a group of transcription factors, which are also involved in the control of Gl biosynthetic genes (Gigolashvili et al., 2007a,b, 2008; Hirai et al., 2007; Sønderby et al., 2007; Malitsky et al., 2008; Sønderby et al., 2010; Li et al., 2013; Jensen et al., 2014). *AtSOT16* was significantly down regulated in *myb34 myb51 myb122-2* triple mutant, revealing the specific control of these transcription factors (Frerigmann and Gigolashvili, 2014). Furthermore, *MYB51*, an indolic Gl metabolism specific transcription factor, was found to be down regulated in the dark, resulting in repression of indolic dsGl specific *AtSOT16*. Another indolic Gl transcription factor, *MYB34*, was up regulated after re-illumination (Celenza et al., 2005). This was not the case for *MYBs* controlling aliphatic Gl biosynthesis. It was concluded that *MYB* factors controlling biosynthesis of indolic Gl have a specific function in light regulation of their target gene, unlike the aliphatic group of *MYB* (Huseby et al., 2013).

Even so, the interaction and hierarchy of HY5 and MYBs still remains unclear. *AtSOT16*, *AtSOT17*, and *AtSOT18* were also up regulated in *apk1 apk2* double mutants. Hence, a reduction in PAPS supply and therefore reduction in Gl concentration leads to an up regulation *dsGl SOTs* (Mugford et al., 2009).

The expression of the twelve putative *dsGl SOTs* in *B. rapa* was investigated by qRT-PCR (Zang et al., 2009). Two genes are paralogs of *AtSOT16*, one of *AtSOT17*, and ten of *AtSOT18*. Generally, *BrSOT16s* were most strongly expressed, followed by *BrSOT18s* and then the *BrSOT17s*. With the exception of one *BrSOT18*, all of them were expressed in all examined tissue types. One *BrSOT16* was expressed in all tissue types, except in the stamen, while the other one was strongly expressed in the stamen, but weakly in the floral bud and carpel. Some *BrSOT18s* were strongly expressed in the carpel and others in the stamen. Hence, the expression was not tissue-specific, but there was great variation in between tissue types. The expression of some *BrSOT18s* was developmentally regulated, but not of *BrSOT16s*. Again it was concluded that the expression could influence the Gl content, since SOTs play a crucial role in Gl biosynthesis.

The *TPST* gene is expressed in the whole plant, which was shown by analyzing *A. thaliana TPST-GUS* transformants, but especially strong in the root apical meristem and in the lateral root primordial and vascular tissues (Komori et al., 2009).

Expression of the 35 *O. sativa SOTs* was investigated by microarray database analysis (Chen et al., 2012). The overall expression was reported to be considerably low. Low expression levels were in the apical meristem and young leaves. Higher expression was found in the stigma, ovary and roots. Treatment with IAA and BAP led to up and down regulation of several *SOTs* also with differences in respect to tissue types and seedlings age. Furthermore, expression of eleven *SOTs* reacted to abiotic stress, such as high and low temperatures and dehydration. It was concluded that the individual responses of *SOTs* indicate functions in stress response and plant development.

Overall, *SOT* expressions suggest functions in plant defense, stress response, signaling and developmental regulation. Sulfation can either lead to activation or deactivation of the according substrate. *SOT* expression takes place basically in all organs and many stages in plant development. Interestingly, all *SOTs* studied so far, were induced by several conditions or stress signaling compounds, indicating a general stress response. Additionally, in the case of *dsGl AtSOTs* and *AtSOT15*, a diurnal and circadian control was detected. It seems plausible that this could be the case for other *SOTs*, too.

## WHAT IS KNOWN ABOUT THE REACTION MECHANISM OF SULFOTRANSFERASES

So far, the reaction mechanism of plant SOTs remains largely unknown. Kinetic and inhibition studies of a flavonol 3′-SOT from *F. chloraefolia* A. Gray led to the hypothesis of an ordered Bi-Bi mechanism (Varin and Ibrahim, 1992). However, the few conducted experiments are not sufficient enough for a definite conclusion.

More information is available about human SOTs. By pre-steady state binding studies, isotopic trapping, quenched-flow, and classic inhibition studies, Wang et al. (2014) completely solved the kinetic mechanism of the human SOT SULT2A1. SULT2A1 sulfates dehydroepiandrosterone and regulates binding of steroids to their receptors and detoxifies steroid-like xenobiotics. The according mechanism was found to be rapid equilibrium random. In this mechanism, substrates are bound and products are released in a random order. The ligands are bound in separate binding sites and released independently of the presence of its partner, hence without contribution of sulfuryl-group interactions. Ligand-binding rate constants also indicated that ligand-protein interactions, which enable the chemical reaction, are either established prior to addition of the second substrate and/or they are engaged as the system moves toward the transition state. Furthermore, it was shown that the release of the PAP nucleotide is the rate-determining step of the reaction. Substrate inhibition was explained by trapping of PAP in a dead end complex (enzyme with bound PAP and substrate), which decreases the release of PAP. Since closely related enzymes often share the same mechanism, it was speculated that this could also be the case for other human SOTs. Anyhow, this cannot be done for plant SOTs without further experimental analysis. This is already illustrated, when regarding that SULT2A1 is a half-site reactive dimer, while yet investigated plant SOTs are monomers.

The mechanism of a monomeric SOT Stf0 from *Mycobacterium tuberculosis* was analyzed by electrospray ionization mass spectrometry and Fourier transform ion cyclotron resonance mass spectrometry (Pi et al., 2005). Stf0 forms trehalose sulfate, which is the core disaccharide of the potential virulence factor sulfolipid-1. Interestingly, the results also indicated a rapid equilibrium random mechanism, at which the sulfuryl group is transferred in the ternary complex. Again, there is one binding site for products and one independent binding site for substrates. Results also indicated that PAPS binding was competitively inhibited by PAP.

Further studies of non-herbal SOTs, human estrogen SOT (Zhang et al., 1998) and insect retinol dehydratase (Vakiani et al., 1998), also indicated random Bi-Bi mechanisms. So far, only for a *Rhizobium meliloti* NodH SOT a hybrid random ping-pong mechanism was suggested (Pi et al., 2004). Therefore, the investigation of the complete kinetic mechanisms of plant SOTs remains an interesting task, which could also give new insights of the overall evolution of SOTs.

## HOW TO IDENTIFY THE SUBSTRATE SPECIFICITY?

### CHANCES AND RESTRICTION OF MODELING

Simple online tools like SWISS-MODEL do not lead to satisfying Z-scores and therefore unreliable models. However, Cook et al. (2013) generated significant models of human SOTs by using more advanced programs such as MODELLER, GOLD, GROMACS, and AMBER. Models of human SOTs SULT1A1 and SULT1A2, which are Phase II detoxifying enzymes, were used for *in silico* docking studies. As substrates, 1455 small molecule drugs were tested. For SULT1A1, 76 substrates were predicted, of which 53 were already known substrates. Of the remaining 23 putative substrates, 21 were tested in enzyme assays and all of them were accepted as substrates. Of 22 predicted substrates for SULT2A1, eight were not previously mentioned in literature. Enzyme assays were carried out with four of the eight newly identified substrates, and all of them were accepted as substrates. For both SOTs neither a single false positive nor a false negative prediction occurred. Furthermore, 136 SULT1A1 and 35 SULT2A1 inhibitors were predicted. Two of those were exemplary tested in classical inhibition studies and both showed inhibitory effects.

But can these techniques be transferred to plant SOTs? Principally they could be transferred to plant SOTs, but it has to be kept in mind that SULT1A1 and SULT1A2 are extensively studied SOTs. Building reliable models for *in silico* docking studies requires knowledge about structure and mechanism of the protein. For example, SULT1A1 and SULT1A2 have a site cap, which regulates substrate specificity (Cook et al., 2012). This also had to be considered when generating the *in silico* models. Furthermore, it was shown that human SOTs have a high plasticity (Allali-Hassani et al., 2007; Berger et al., 2011) and that PAP binding leads to dramatic conformational changes, such as pre-formation of the acceptor binding pocket (Bidwell et al., 1999; Dajani et al., 1999; Berger et al., 2011). Hence, without prior structural knowledge about at least some of the plant SOTs, *in silico* modeling is still restricted. Nevertheless, *in silico* modeling of SOTs is a promising approach, especially because of the limitations in substrate identification based on phylogenetic analyses.

### *A. thaliana* AS A MODEL PLANT – SUITED FOR THE ELUCIDATION OF ALL SOT FUNCTIONS?

Elucidation of all SOT functions in *A. thaliana* as a model plant is difficult for several reasons. Until now, ten out of 22 identified putative *A. thaliana* SOTs have been enzymatically characterized *in vitro*. The identified substrates were peptides, flavonoids, brassinosteroids, GIs, hydroxyjasmonate, and salicylic acid. As discussed before, prospects of phylogenetic analyses are very limited for SOTs. Already small changes in the sequence can lead to wide variations in substrate specificity. Even reliable predictions of yet uncharacterized SOTs in the organism *A. thaliana* are not possible. Therefore, reliable function prediction of SOTs in other plant species on the basis of *A. thaliana* sequences seems very unlikely.

Even when comparing SOTs that use the same class of substrates from different plant species, not only differences in kinetic values, but also variation of specificity toward different substrates and specific hydroxyl groups are noticed. For example, flavonol SOTs (AtSOT5, AtSOT8, AtSOT12, AtSOT13) from *A. thaliana* prefer kaempferol or flavonol glycosides as substrate and sulfate the hydroxyl groups at 3- and 7-position (Hashiguchi et al., 2013, 2014). But flavonol SOTs from *F. chloraefolia* and *F. bidentis* (L.) Kuntze prefer quercetin as substrates and sulfate at 3′- and 4′-position (Varin et al., 1992, 1997b). Furthermore, all so far characterized *A. thaliana* SOTs sulfate a broad range of substrates. Most are functionally and biochemically related, but for example in case of AtSOT12, substrates with a wide range of biological functions are accepted as substrates.

Another difficulty is that SOTs are part of secondary metabolism and therefore fulfill species-specific functions. Hence, it is unlikely that all types of SOTs occur in *A. thaliana*. This is supported by the Pfam database research described in chapter 3.1. In *A. thaliana*, only the TPST contains a *Sulfotransfer_2* domain (PF03567). The remaining AtSOTs all contain a *Sulfotransfer_1* domain (PF00685), while the *Sulfotransfer_3* (PF13469) and *Gal-3-O_sulfotr* domain (PF06990) are only present in algae. *Arylsulfotran_2* domain (PF14269) is only found in a single *Ricinus communis* sequence. In addition, *SOT* homologues in different plant species differ in their number of paralogs. For example, there were nine homologues of *AtSOT18* found in *B. rapa* (Zang et al., 2009), which could differ in their characteristics.

All in all, it remains an important future task to clarify the biological functions and characteristics of the remaining *A. thaliana* SOTs, not only by *in vitro* enzymatic assays, but in consideration of mutation, expression and localization studies, as well as metabolomics. Findings could at least be partly transferred and give valuable hints about specific SOTs in other species. Since *A. thaliana* is the most studied plant, complete characterization of all AtSOTs could also give more information about the connection of primary and secondary metabolisms in plants in general.

## FUTURE CHALLENGES

Plant SOT research still remains a biological field with many open questions, especially in comparison with mammalian SOTs. In the model organism *A. thaliana*, only ten out of 22 SOTs have been enzymatically characterized *in vitro* so far. In many of these cases, the *in vivo* function is not elucidated yet. Some compounds, which were found to be sulfated by SOTs *in vitro*, could not be detected *in vivo*, as it was the case for sulfated glucoside flavonoids, sulfated by AtSOT8 (Hashiguchi et al., 2014). Furthermore, the function of sulfation or the sulfated compound is often not completely understood. Hence, for a deeper understanding it is advisable to follow *in vitro* enzymatic characterization with mutation, expression and localization studies.

For the remaining twelve putative *A. thaliana* SOTs, disregarding the pseudogene *AtSOT2*, no accepted substrates have been identified so far. Due to the enormous number of putative substrates and the restricted reliability of phylogenetic analyses, complete functional elucidation of all AtSOTs is an ambitious goal. Hence, recombinant expression and offering randomly chosen substrates seems like looking for a needle in a haystack. A more promising approach could be to feed wild-type and mutant plants with ^35^S, followed by mass spectrometry analysis. This can facilitate the identification of newly sulfated compounds *in vivo.* Next steps could be the isolation of these compounds, depending on its availability and chemical properties. If possible, the compounds could be bound to a column and used for affinity chromatography of total protein preparations. This would be a very systematic approach and was already partly used for the successful identification of TPST (Komori et al., 2009).

Especially in pharmaceutical research, *in silico* analysis has become a powerful tool (Song et al., 2009). With the help of three dimensional structures of the target molecules, computational drug design becomes more and more promising. The three dimensional structure of one *A. thaliana* SOT (AtSOT12) has already been solved, but without including substrates into the crystals (Smith et al., 2004). A deeper understanding could be reached with the help of more solved structures with and without substrates and with additional knowledge about the enzymatic mechanism. Definitely identified binding sites, combined with protein modeling could give more specific hints about putative substrates of SOTs.

Another interesting field would be the elucidation of SOTs from more plant species, especially highly specialized ones. In order to cope with additional stress, plants growing in challenging environments often biosynthesize specific compounds. Its properties are often promising from a biological point of view, for a better understanding of stress response, but also interesting for medical or biotechnological applications. Zosteric acid [*p*-(sulfo-oxy) cinnamic acid] from the seagrass *Zostera marina*, for example, has anti-fouling properties (Newby et al., 2006). In a patent, SOT involvement in biosynthesis was suggested, but not proven yet (Alexandratos, 1999). Additionally, in *Zostera*, *Halophila,* and *Thalassia* seagrass, the existence of sulfated flavones was indicated (Harborne and Williams, 1976). Furthermore sulfated polysaccharides were detected in seagrass (Aquino et al., 2005, 2011), freshwater plants (Dantas-Santos et al., 2012) and algae (Ngo and Kim, 2013). While the sulfation of polysaccharides is well-studied in humans (Kusche-Gullberg and Kjellén, 2003), no polysaccharide SOTs have been studied in plants yet. It is hypothesized that sulfated polysaccharides modify the cell wall in halophytes in order to increase salt tolerance (Aquino et al., 2005). They are also interesting for human nutrition and pharmaceutical products, because of their antioxidant, anti-allergic, anti-human immunodeficiency virus, anti-cancer and anticoagulant properties (Ngo and Kim, 2013).

Overall, substrate specificities, regulations, and catalytic mechanisms of plant SOTs are still poorly understood. Considering the large number of possible functions, further research on these enzymes remains a challenging field.

## Conflict of Interest Statement

The authors declare that the research was conducted in the absence of any commercial or financial relationships that could be construed as a potential conflict of interest.
